# Risks and opportunities: cholangioscopy-assisted strategic guidewire manipulation for the management of occlusive anastomotic stricture after liver transplantation

**DOI:** 10.1055/a-2743-2315

**Published:** 2025-12-03

**Authors:** Shanbin Wu, Jielei Li, Jie Ni, Yan Zhang, Guodong Li, Haiyan Dong, Wei Li

**Affiliations:** 1 66310 Department of Gastroenterology, The First Affiliated Hospital of Shandong First Medical University and Shandong Provincial Qianfoshan Hospital, Jinan, China; 2 66310 Department of Pediatrics, The First Affiliated Hospital of Shandong First Medical University and Shandong Provincial Qianfoshan Hospital, Jinan, China

An anastomotic stricture is a common complication after liver transplantation, which can lead to liver dysfunction and cholestasis, and requires prompt intervention 1 2 . Endoscopic retrograde cholangiopancreatography (ERCP) with balloon dilation and stent placement is the preferred treatment 1 2 . In the case of severe stenosis, cholangioscopy-assisted guidewire manipulation may be crucial 3 .

This report describes a case of a near-occlusive anastomotic stricture after liver transplantation, in which cholangioscopy-assisted guidewire manipulation enabled successful passage across the stricture and stent placement ( Video 1 ). A 51-year-old man, 17 months after liver transplantation, was admitted with a 2-week history of progressive elevation of aminotransferases, alkaline phosphatase, and bile acids, along with mild hyperbilirubinemia. Magnetic resonance cholangiopancreatography revealed a marked anastomotic stricture ( Fig. 1 ). During ERCP, the guidewire repeatedly looped at the stricture. Neither the contrast agent nor the guidewire could enter the intrahepatic bile ducts ( Fig. 2 ). Cholangioscopy showed an occlusive anastomotic stricture. Under cholangioscopic vision, attempts to pass the guidewire through two suspected orifices ( Fig. 3 ) were unsuccessful in crossing the stricture. The wire was then reversed, and its stiff segment was carefully advanced under direct vision into a suspected orifice. After feeling a breakthrough, fluoroscopy confirmed that the guidewire had passed the stricture. Then, the guidewire was withdrawn, and the bile outflow was observed. The soft tip was reintroduced and successfully advanced into the intrahepatic ducts. Balloon dilation was performed, and two plastic stents were placed. Post-procedure, liver biochemistry improved significantly. This case demonstrates the strategic use of different guidewire segments in managing severe biliary strictures after liver transplantation, while acknowledging the potential risk of portal vein injury. Since portal vein bleeding is usually self-limiting, the potential benefits to the patient outweigh the risks, making the procedure worthwhile to attempt.

After conventional methods failed, the stiff segment of the guidewire was carefully advanced under direct cholangioscopic visualization to cross the stricture, enabling successful stent placement.Video 1

**Fig. 1:**
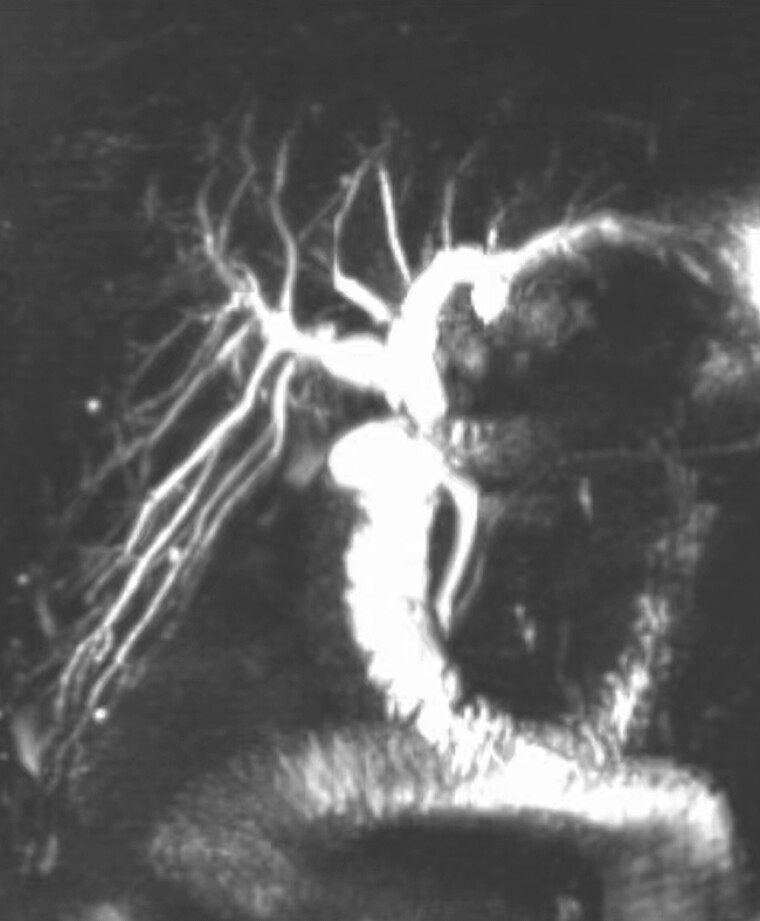
Pre-procedure MRCP showing an anastomotic stricture with upstream extrahepatic and intrahepatic bile duct dilatation. MRCP, magnetic resonance cholangiopancreatography.

**Fig. 2:**
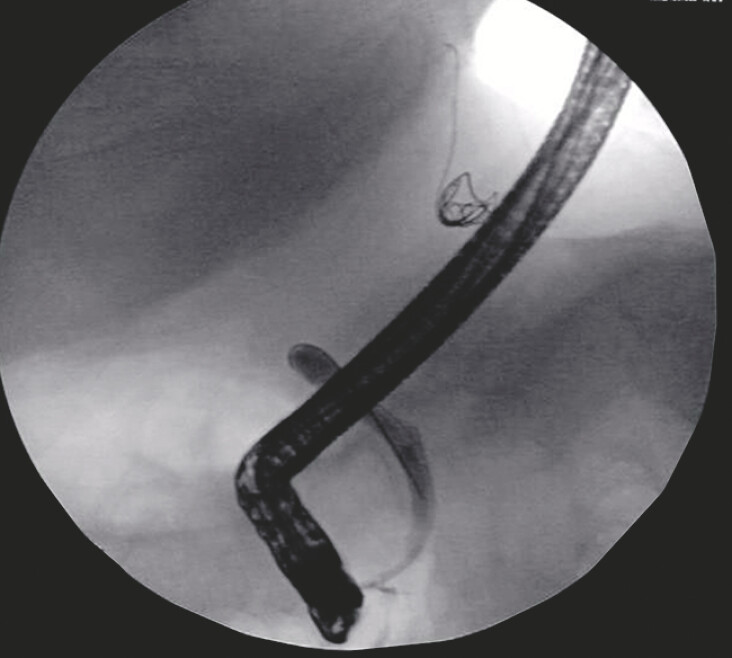
Neither the contrast agent nor the guidewire could pass the stricture to enter the intrahepatic bile ducts.

**Fig. 3:**
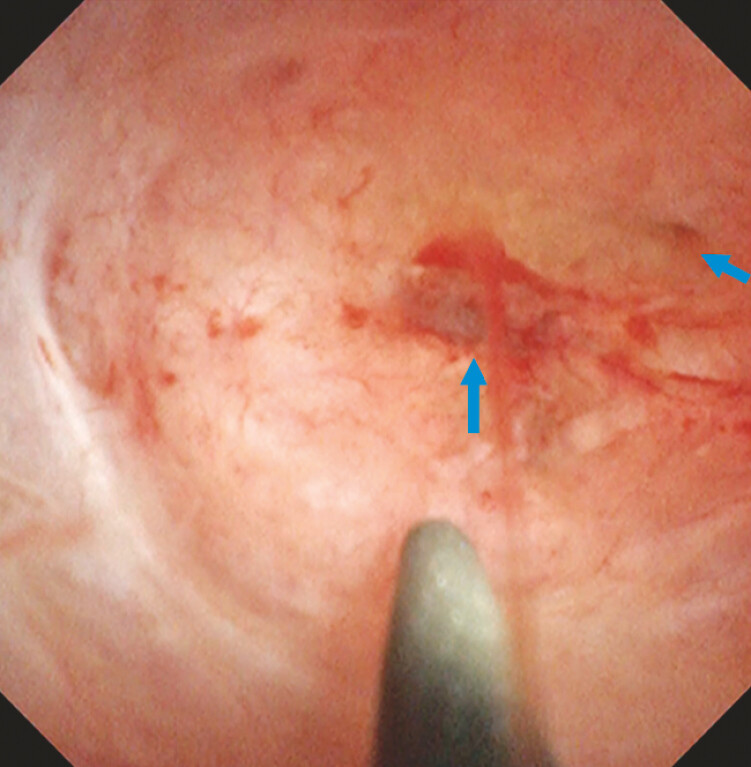
Cholangioscopy revealing an occluded anastomosis; two depressions are seen as suspected orifices (blue arrows).

Endoscopy_UCTN_Code_TTT_1AR_2AG

## Notes

Endoscopy E-Videos https://eref.thieme.de/e-videos
*E-Videos* is an open access online section of the journal *Endoscopy* , reporting on interesting cases and new techniques in gastroenterological endoscopy. All papers include a high-quality video and are published with a Creative Commons CC-BY license. Endoscopy E-Videos qualify for HINARI discounts and waivers and eligibility is automatically checked during the submission process. We grant 100% waivers to articles whose corresponding authors are based in Group A countries and 50% waivers to those who are based in Group B countries as classified by Research4Life (see: https://www.research4life.org/access/eligibility/ ). This section has its own submission website at https://mc.manuscriptcentral.com/e-videos .
